# Balancing trade-offs between nutritional quality, consumer acceptability and climate impact across a spectrum of chili con carne formulations: from plant-based to hybrid

**DOI:** 10.3389/fnut.2025.1716322

**Published:** 2025-11-21

**Authors:** Mari Wollmar, Anna Post, Maja Elf, Josephine Ingridsdotter, Mia Prim, Agneta Sjöberg

**Affiliations:** Department of Food and Nutrition and Sport Science, University of Gothenburg, Gothenburg, Sweden

**Keywords:** sustainable public meals, absorbable iron, meal development, plant-based, taste

## Abstract

**Introduction:**

Transitioning to sustainable food consumption, through nutritious and low carbon diets, is essential to address climate and health challenges. Current trends indicate an increase of plant-based meals in schools. However, these climate-friendly options are not consistently well-received by students and often fail to meet dietary iron requirements, particularly for adolescent girls. This study aims to develop a methodology for creating sustainable school meal recipes that balance absorbable iron, carbon footprint, and taste preferences.

**Methods:**

The methodology involves iterative recipe development, including cooking elaborations and consumer evaluations. A chili con carne recipe was selected as the test dish where various plant-based and meat hybrid alternatives were assessed. Absorbable iron was calculated using the Hallberg and Hulthén algorithm, factoring in inhibitors and enhancers. Consumer evaluations were conducted in two rounds with university staff and students as a methodological validation step, involving sensory assessments and preference tests.

**Results:**

The study found substantial differences between total iron content and absorbable iron, with plant-based recipes (Soy1) achieving only 35% of the required absorbable iron for high-need teenage girls despite meeting total iron requirements. Hybrid recipes incorporating both meat and plant-based ingredients showed better iron bioavailability and were more acceptable to consumers. Optimized recipes reduced carbon footprint by 16–84% compared to the original recipe, with hybrid recipes (Beef/Soy and Beef/Lentils) achieving 37–39% reductions while maintaining adequate absorbable iron levels (0.42–0.56 mg per meal vs. 0.44–0.66 mg target range).

**Discussion/Conclusion:**

The findings suggest that hybrid recipes can effectively balance absorbable iron, carbon footprint and taste preferences to promote sustainable and healthy eating habits among adolescents. Validation with the target adolescent population in school settings is recommended as the essential next step.

## 1 Introduction

The shift toward plant-based diets has emerged as a key strategy for addressing both environmental sustainability and human health, as advocated by the 2030 Agenda for Sustainable Development (1) and numerous research studies (2–5). The latest Nordic Nutrition Recommendations (NNR2023) reflect this shift by promoting plant-based diets and reducing animal product consumption to enhance health and sustainability (Nordic Council of Ministers 2023). However, in the eagerness to climate-adapt meals mineral bioavailability, as well as taste preference may be disregarded. Plant-based diets often present challenges in iron absorption compared to diets that include meat. This is primarily because plant foods lack heme iron, which is found in meat, poultry and fish and is more readily absorbed by the body (6).

Plant-based alternatives may seem like adequate iron sources due to their usually high total iron content, but absorbable iron is often overlooked. While promising for low carbon meals, they need nutritional improvements, particularly for iron. Soy protein isolates and concentrates are popular ingredients in plant-based foods but are also high in phytates, which can inhibit iron absorption (7–9). Phytate binds with iron and forms insoluble complexes in the digestive tract, making it difficult for the body to absorb the iron effectively (9). Strategies to counter phytate’s effect on iron absorption include consuming vitamin C-rich foods and/or adding small amounts of meat, which enhances non-heme iron absorption from the meal (7).

The recent update to the EAT-Lancet Commission’s Planetary Health Diet (2024) reaffirms the need for dietary transitions toward plant-forward food systems to achieve both human health and environmental sustainability goals (4). While the framework emphasizes reducing red meat consumption and increasing legume intake, the Commission acknowledges that meeting iron requirements through predominantly plant-based diets may be challenging without supplementation (4). This highlights the importance of meal optimization and product innovation strategies that can enhance iron bioavailability from plant-based and hybrid recipes.

Adjusting the iron content in the planetary health diet requires reducing phytate levels to effectively address iron deficiencies (10). This is particularly important for teenage girls and women of childbearing age (11, 12). Iron deficiency is prevalent among teenage girls (13, 14) and can severely impact physical health, cognitive development, and overall well-being, particularly in adolescents (15, 16).

When developing school lunch recipes, it is crucial to consider both taste preference and absorbable iron. In Sweden, public school meals are free for all students, funded by the government, and aim to provide nutritious, balanced meals that meet national dietary guidelines. Swedish schools serve approximately 1 million meals daily, making school lunches an ideal platform for promoting sustainable eating habits (17). Sensory evaluations and preference tests play a key role in identifying the factors that drive food acceptance (18). Plant foods, especially phenol-rich ones, tend to have stronger sour and bitter tastes compared to meat. This can make plant foods more challenging for some consumers to accept compared to meat products (19).

Therefore, encouraging the adoption of plant-based diets during school years can be beneficial for fostering long-term, healthy, and climate-conscious food choices by gradually incorporating more plant-based ingredients (20). In Swedish school meals, soy-based meat substitutes are widely used in vegetarian meals due to their compatibility with meal production techniques (21). Another option is using meat extenders like lentils, soy, and pea products in familiar dishes, either by reducing or substituting animal-based components. Hybrid recipes that combine plant-based ingredients with meat may not instantly shift attitudes but offer promising opportunities for reducing carbon footprint and improving iron bioavailability.

Given these challenges, there is an urgent need for a systematic approach to recipe development that can simultaneously address nutritional adequacy, environmental sustainability, and consumer acceptance in school meal planning. This study develops a methodology for school meal planners, inspired by the culinary funnel, an iterative process of recipe improvement through cooking elaborations and consumer evaluations, as suggested by Westling et al. (18). Our study aims to develop and validate a systematic methodologythat ensure that teenage girls meet their absorbable iron needs, while also considering carbon footprint and taste preferences. By focusing on both total and absorbable iron in our nutritional assessment, we strive to create recipes that are tasty, nutritionally beneficial, and sustainable. Therefore, the objective of this study is to develop and validate a systematic methodology for creating sustainable school meal recipes that optimize the balance between absorbable iron content, carbon footprint reduction, and consumer acceptance, specifically targeting the nutritional needs of adolescent girls. This methodology employs iterative recipe development through cooking elaborations and consumer evaluations to create hybrid recipes that can effectively bridge the gap between nutritional adequacy and environmental sustainability in school meal planning.

## 2 Materials and methods

### 2.1 Recipe development

The study employed a modified culinary funnel approach, beginning with an established base recipe rather than a specific crop variety. This adaptation preserved the methodological framework’s systematic nature while repositioning the entry point. The process commenced with selection of the foundational recipe, followed by controlled variation of ingredients, preparation techniques, and cooking parameters. Each iteration underwent sensory evaluation to identify optimal modifications. The recipe was then tested across diverse culinary applications through a series of elaborations, with consumer feedback collected at key stages. This iterative development process allowed findings from each application to inform subsequent refinements, creating a continuous feedback loop that maximized the recipe’s versatility while maintaining its essential character. The methodology prioritized both sensory quality and functional adaptability throughout the development cycle.

The recipe going into the culinary funnel was a chili con carne chosen from a national survey (50) [unpublished], the recipe was retrieved from a participating municipality. Chili con carne served as the test dish because it worked well as a methodological example. The test dish offered possibilities considering all our areas of interest which were securing adequate iron intake, reducing carbon footprint, working with meat-alternatives and improving the sensory appeal. The optimization process included multiple recipes with plant-based meat substitutes, lentils, or legumes, mushrooms, seasoning and adjustments for energy content alignment. Standards set by the Swedish national food agency’s guidelines for school meals (22). The guidelines have recommendations for energy per portion, total fat and fatty acids, carbohydrates, protein, fiber, vitamin C, vitamin D, folate, iron and salt (22). This guideline for absorbable iron was informed by the iron requirements for teenage girls aged 11–14 and 15–17 years, as outlined in the Iron background article for NNR2023 (12). To meet the iron needs of the median group of girls, the amount of absorbable iron in each school lunch should be 0.44 mg/school lunch. To meet the nutritional needs of 95% of girls, the absorbable iron content must be to 0.66 mg/ school lunch (12). Calculations assumed that a school lunch should provide 30% of the daily requirements/needs (22). All recipes can be found in Supplementary material 1.

According to the National guidelines for school meals, nutritional calculations should reflect a complete meal offer. For this reason, a standard is used that includes other components. In our study, recipes were calculated using a standard consisting of vegetables (138 g), crispbread (1 slice), spread (10 mg 40% fat) and milk (1 dL, 1.5% fat). Calculations were made both with and without milk.

### 2.2 Calculation of absorbable iron and CO_2_e

Absorbable iron was calculated using the Hallberg and Hulthén (7) algorithm, factoring in inhibitors (phytate phosphorus, calcium and soy protein) and enhancers (meat factor and vitamin C) and amount of heme vs. non-heme iron, as well as interactions between inhibitors and enhancers. The choice of algorithm was based on previous literature, where it was found to be the most accurate for evaluation absorbable iron among women of childbearing age and omnivore dietary patterns (6, 23). Further, the findings of Hoppe et al. (24) using the algorithm highlights the critical role of bioavailability in assessing iron adequacy in adolescent diets.

The inclusion of soy protein as an inhibitor is based on information that soy protein significantly reduces iron absorption, even without phytate, indicating it has a strong inhibitory effect on its own (7, 25). The amount of soy protein in grams was calculated to 26 g/100 g based on information from a product commonly used in Swedish public meals. The calculation was based on the absorption rate of a teenage girl with low or empty iron stores (serum ferritin <15 μg/L) (7). A serum ferritin level below 15 μg/L suggests iron deficiency (26). This is lower than mean serum ferritin of 31 μg/L in Swedish adolescent girls (13). A serum ferritin of 15 μg/L (7) was used to avoid unnecessarily low estimates of absorbed iron since iron absorption is upregulated in subjects with low or empty iron stores.

Information regarding calcium content was sourced from the Swedish food composition database (27) while phytate content was obtained from the literature. Most products were referenced from (7) and the book Food Phytates (9). For new plant-based meat alternatives, data were taken from Mayer-Labba et al. (8), and for bulgur, from Ertaş (28). Detailed sources for each ingredient’s phytate content are provided in Supplementary material 2.

Phytate content values (mg/100 g) for all ingredients were obtained from published literature rather than through direct laboratory analysis. Values were sourced from (7–9, 28). These literature-based phytate values were selected based on comparable analysis methods. Values used in this study are presented in Supplementary material 2.

#### 2.2.1 Sensitivity analysis for absorbable Iron calculations

To assess the robustness of absorbable iron calculations given the variability in phytate and soy protein content across commercial products, we conducted sensitivity analyses varying key parameters: 1. Soy protein content: Varied from 20 to 32 g/100 g (±23% from baseline 26 g/100 g) to reflect variation across commercial soy mince products 2. Phytate content: Varied by ±20% from literature values for all phytate-containing ingredients, with phytate content being the most sensitive parameter (detailed results in Supplementary material 2). Importantly, the ranking of recipes by absorbable iron content remained consistent across all sensitivity scenarios, confirming that our conclusions regarding recipe adequacy for meeting iron requirements are robust to uncertainty in input parameters. For all main analyses, we used the baseline parameters (26 g/100 g soy protein, literature phytate values, 15 μg/L serum ferritin) representing the target population of teenage girls with high iron needs.

#### 2.2.2 General nutrition

General nutrition was evaluated using Dietist Net nutrition calculation program version 24.01.03 (Kost- och Näringsdata AB, Bromma, Sweden). Carbon footprint calculations were conducted using the RISE climate database (29) integrated as a plug-in within DietistNet where the mean value for each product was used. In the absence of guidelines for the CO_2_e of school meals, we calculated the lowest possible CO_2_e based on the available products in public food service procurement and ensuring sufficient bioavailable iron in the meal. All developed recipes were compared to the original when calculating changes in CO_2_e.

### 2.3 Participants

Participants for the consumer evaluation, conducted in a test environment, were recruited from students and staff at the University of Gothenburg. All participants received written and oral information detailing the project, participation conditions, and data handling procedures. All participants signed a consent form affirming their understanding of the project, and participation and were given the opportunity to seek clarification. Additionally, participants confirmed that they were omnivores and attested to the absence of food sensitivities or allergies. The study is approved by the Swedish Ethical Review Authority (No. 2022–04834-01).

Round 1 consisted of 48 participants aged 21 to 65 years, 32 women, 15 men, and 1 gender-undisclosed participant. Evaluations occurred on five occasions, with 7 to 12 participants per session. Round 2 involved 54 participants aged 19 to 66 years, 32 women, 21 men, and 1 gender-undisclosed participant. These evaluations were conducted on four occasions, with 11 to 21 participants per session. Ten participants attended both test rounds, resulting in a total of 92 unique participants and 102 evaluations. Detailed participant characteristics are provided in Supplementary material 3.

### 2.4 Consumer evaluations

Consumer evaluations were conducted in two test rounds: round 1 in spring 2023 and round 2 in spring 2024. In each test round, multiple variants of chili con carne were evaluated—four in round 1 and three in round 2. The evaluation session lasted between 15 and 25 min for each group. The test samples were prepared in the kitchen adjacent to the dining area, with dishes kept warm for 30 minutes to 1 h before serving. Each sample had two identification numbers and was presented in random order. Samples were served in clear 0.15 L plastic cups suitable for visual examination. All samples were presented on a white rectangular plate, accompanied by a glass of water and two wheat crackers for palate cleansing between tastings. The process began with the consumers assessing the hedonic value of each sample using a 9-point hedonic scale, where 1 indicated ‘extremely dislike,’ 5 indicated ‘neither dislike nor like,’ and 9 indicated ‘extremely like’ (30, 31). After this, the consumers rated their liking of intensity of the product’s sensory attributes “amount of beans,” “richness,” “spiciness,” “seasoning” and “saltiness.” The term ‘richness’ in this context refers to the perception of savory, full-bodied flavor intensity and mouthfeel commonly associated with umami taste and fat content. Participants were asked to rate the richness of each sample, with the understanding that richness encompasses the depth and complexity of savory flavors. For this, a 5-point JAR-scale was used (32). Participants provided insights into preferences and potential improvements through open-ended questions. At the end of the questionnaire, they were instructed to rank the samples, starting with their preferred sample.

The results were analyzed after round 1 of consumer evaluations. The analysis served as the basis for the next round of recipe selection, cooking elaboration, and consumer evaluation. In round 2, all recipes were energy-adjusted with rapeseed oil to meet the stated energy requirement for girls and boys age 13–15 years by the standards set by The Swedish national food agency (2019).

In Round 1 a flavor base was decided that consists of the aromatic vegetables (carrot, celeriac, onion), spices, and other seasonings that provide the foundational flavor profile, while the protein component (beef, soy mince, or lentils) was varied

In the second round, a comparative evaluation was performed between the hybrid recipes (Beef/Soy2 and Beef/Lentils2) and a meat-only recipe (Beef2, containing 50 g ground beef). All three recipes utilized the improved flavor base developed in Round 2, which included doubled cumin and sun-dried tomato paste.

### 2.5 Data analysis

To analyze the hedonic data from participants, data from the 9-point hedonic scale was checked for normality using the Shapiro–Wilk test. The results revealed a non-normal distribution. Consequently, we employed non-parametric tests for analysis. Friedman’s ANOVA was used to determine if there were any significant differences in liking between variants, with a significance level of *p* < 0.05. When significant differences were detected, post-hoc pairwise comparisons were conducted using Wilcoxon signed-rank tests with Bonferroni correction to control for multiple comparisons (detailed pairwise comparison results are provided in Supplementary material 3). Effect sizes were calculated using Kendall’s W to quantify the magnitude of differences in preference ratings.

Ten participants attended both evaluation rounds. To address potential data dependency from these repeated participants, we conducted sensitivity analyses by re-running all analyses excluding these individuals. Results showed no substantial differences in hedonic ratings, recipe rankings, or statistical conclusions, indicating that the inclusion of repeated participants did not bias our findings. Additionally, the primary comparisons were made within each round rather than between rounds, minimizing the impact of this dependency. Therefore, all reported analyses include data from all participants.

Sample sizes (*n* = 48 for Round 1, *n* = 54 for Round 2) were determined based on practical considerations for sensory testing and previous literature suggesting that 40–60 participants provide adequate discrimination power for hedonic preference tests (32). While no formal power calculation was conducted *a priori*, the consistency of our findings across both rounds, the lack of significant differences despite adequate sample sizes, and the clear patterns in preference rankings support the adequacy of these sample sizes for detecting meaningful differences in acceptance.

A modified penalty analysis was performed to identify attributes that driving decrease in overall liking. For each JAR (Just-About-Right) attribute (beans, richness, spiciness, seasoning, saltiness), responses on the 5-point scale were categorized as” too little” (ratings 1–2),” just-about-right” (rating 3), or” too much” (ratings 4–5) (30, 32). The mean drop in liking was calculated as:

Mean drop = (Mean liking for JAR group) - (Mean liking for” too little” or” too much” group).

Only attributes where ≥20% of respondents indicated deviation from JAR were considered actionable, following recommendations by Ares et al. (30). Statistical significance of the mean drop was assessed using independent t-tests comparing the JAR group versus the non-JAR group for each attribute (*α* = 0.05). It is important to note that despite penalty analysis in Round 1 suggesting increased salt could improve liking for Beef/Soy1 (*p* = 0.04, mean drop = 0.8 points on the 9-point scale), salt content was not increased in subsequent iterations. This decision prioritized adherence to Swedish National Food Agency nutritional guidelines limiting salt to 1.8 g per school lunch for children aged 13–15 years (22) over marginal improvements in hedonic ratings. Statistical analyses were conducted using XLstat (Addinsoft, New York, United States) for basic statistics and penalty analysis and to conduct post-hoc tests, while R Statistical Software (v4.1.2) was used to visualize the result of the 5-point JAR scale of the five attributes.

## 3 Findings

Our study assessed absorbable iron, carbon footprint and taste preference in multiple versions of a chili con carne recipe, considering typical complements in school lunches to evaluate a methodology for recipe development. A noteworthy observation was the variance between total iron content and absorbable iron.

### 3.1 Cooking elaborations and consumer evaluations

#### 3.1.1 Round 1 - calculations and recipe selection

Several variants of chili con carne were tested in cooking elaborations based on an original recipe provided by a municipality participating in a national survey (50) [unpublished]. The original recipe contained 50 g of ground beef per portion (Supplementary material 1). Finely chopped and fried carrot (25 g) and celeriac (20 g) were added to all recipes to enhance palatability. Sambal Olek, which was a part of the original recipe, was removed as it was found to add excessive spiciness to recipes where ground beef was replaced with meat substitutes. In preliminary tastings with kitchen staff (n = 6), all 6 testers noted” too spicy” or” overpowering heat” in open-ended comments when Sambal Olek was included with plant-based alternatives, indicating it masked other flavor nuances and created an unbalanced flavor profile. Detailed information on energy and nutrient content can be found in Table 1. Four recipes were after the cooking elaborations and calculations (absorbable iron and CO_2_e) selected for consumer evaluation. The selected variants were Soy1 (50 g of soy mince), Beef/Soy1 (25 g ground beef/25 g soy mince) Beef/Lentils1 (25 g ground beef/15 g dry lentils) and Beef/Beans1 (40 g ground beef/28 g beans). Table 2 presents a comparison of the key factors investigated in this study: absorbable iron per meal, kg CO_2_e per meal and the mean hedonic value. The result of all calculated impact factors is found in Table 2.

**Table 1:** Energy, nutrients, iron and CO_2_e - test round 1.

Nutrient	Soy1 Mean ± SD	Beef/Soy1 Mean ± SD	Beef/Lentils1 Mean ± SD	Beef/Beans1 Mean ± SD	Original Mean ± SD	% of RDI (13–15 years)
a. Chili only (without sides)						
Energy (kcal)	284 ± 5	295 ± 5	305 ± 5	315 ± 5	253 ± 5	39–43%
Protein (g)	15.0 ± 1	16.0 ± 1	16.7 ± 1	17.6 ± 1	16.0 ± 1	60–70%
Fat (E%)	41.5	44.7	37.7	42.7	44.1	25–40% ref
- SFA (E%)	3.8	7.8	7.0	9.6	13.7	≤10% ref
- PUFA (E%)	11.9	10.3	8.7	8.7	7.5	5–10% ref
- MUFA (E%)	23.8	23.8	19.5	21.8	19.6	10–20% ref
Carbohydrates (E%)	37.1	33.2	40.1	34.6	30.3	45–60% ref
Fiber (g)	12.6 ± 1	11.3 ± 1	13.8 ± 1	12.6 ± 1	7.6 ± 0.5	140–153%
Vitamin C (mg)	60 ± 5	60 ± 5	60 ± 5	60 ± 5	72 ± 5	261–313%
Vitamin D (μg)	0.0	0.0	0.0	0.1 ± 0.05	0.5 ± 0.1	0–17%
Folate (μg)	150 ± 10	132 ± 10	144 ± 10	140 ± 10	74 ± 5	82–167%
Iron, total (mg)	3.5 ± 0.3	3.3 ± 0.3	3.8 ± 0.3	3.5 ± 0.3	3.1 ± 0.2	69–84%
b. Complete meal (with bulgur, salad buffet and school-standard milk)						
Energy (kcal)	671 ± 10	682 ± 10	693 ± 10	702 ± 10	640 ± 10	87–95%
Protein (g)	30.1 ± 2	31.1 ± 2	31.8 ± 2	32.7 ± 2	31.1 ± 2	120–131%
Fat (E%)	26.7	28.3	25.5	27.9	27.0	25–40% ref
- SFA (E%)	4.1	5.8	5.5	6.6	7.8	≤10% ref
- PUFA (E%)	7.1	6.5	5.9	5.9	5.2	5–10% ref
- MUFA (E%)	13.6	14.0	12.2	13.3	11.6	10–20% ref
Carbohydrates (E%)	55.1	53.1	55.9	53.2	53.3	45–60% ref
Fiber (g)	22.1 ± 2	20.7 ± 2	23.2 ± 2	22.1 ± 2	17.0 ± 1	189–258%
Vitamin C (mg)	86 ± 7	86 ± 7	86 ± 7	86 ± 7	97 ± 8	374–422%
Vitamin D (μg)	2.5 ± 0.2	2.5 ± 0.2	2.5 ± 0.2	2.6 ± 0.2	3.0 ± 0.2	83–100%
Folate (μg)	245 ± 15	227 ± 15	239 ± 15	235 ± 15	169 ± 10	188–272%
Iron, total (mg)	5.5 ± 0.4	5.3 ± 0.4	5.9 ± 0.4	5.6 ± 0.4	5.2 ± 0.3	116–131%
c. Complete meal (with bulgur, salad buffet, excluding milk)						
Energy (kcal)	612 ± 10	623 ± 10	634 ± 10	644 ± 10	582 ± 10	79–88%
Protein (g)	24.7 ± 2	25.7 ± 2	26.4 ± 2	27.3 ± 2	25.7 ± 2	99–109%
Fat (E%)	28.1	29.9	26.7	29.4	28.5	25–40% ref
- SFA (E%)	3.8	5.7	5.3	6.6	7.8	≤10% ref
- PUFA (E%)	7.8	7.1	6.4	6.4	5.7	5–10% ref
- MUFA (E%)	14.6	15.1	12.1	15.3	12.6	10–20% ref
Carbohydrates (E%)	55.5	53.4	56.4	53.4	53.5	45–60% ref
Fiber (g)	22.1 ± 2	21.1 ± 2	23.2 ± 2	22.1 ± 2	17.0 ± 1	189–258%
Vitamin C (mg)	85 ± 7	85 ± 7	85 ± 7	85 ± 7	96 ± 8	370–417%
Vitamin D (μg)	1.0 ± 0.1	1.0 ± 0.1	1.0 ± 0.1	1.1 ± 0.1	1.5 ± 0.1	33–50%
Folate (μg)	230 ± 15	205 ± 15	217 ± 15	213 ± 15	147 ± 10	163–256%
Iron, total (mg)	5.5 ± 0.4	5.3 ± 0.4	5.9 ± 0.4	5.6 ± 0.4	5.2 ± 0.3	116–131%

Reference values for the energy and nutrient content in an average school lunch, corresponding to 30% of the recommended daily intake (RDI) for girls and boys aged 13–15, according to the Nordic Nutrition Recommendations 2012, as stated by the National guidelines for school meals. SD values are estimated based on typical variation in recipe preparation and ingredient variation. RDI percentages are calculated based on 30% of daily recommended intake for 13–15 year olds. *E% including glycaemic carbohydrates and fiber **Only glycaemic carbohydrates.

**Table 2:** Absorbable iron, carbon footprint, and hedonic ratings for chili con carne recipes.

Recipe	Total Iron (mg)	Heme iron	Non-heme iron	Absorbable iron (mg)	Vitamin C (mg)*	Calcium (mg)	Phytate phosphorous (mg)	CO_2_e (kg/meal)	Hedonic value Mean (SD)
a. Chili only (without sides)									
Soy1	3.7	0.0	3.7	0.19	30	148	157	0.28	6.5 (± 1.20)
Beef/Soy 1	3.5	0.2	3.3	0.37	30	122	115	1.04	7.0 (± 1.08)
Beef/Lentils1	4.5	0.2	4.2	0.52	30	99	105	1.01	6.7 (± 1.08)
Beef/Beans1	3.5	0.3	3.2	0.41	30	170	74	1.48	7.1 (± 1.13)
**Original	3.1	0.38	2.7	0.70	36	84	40	1.79	
Beef/Soy2	4.2	0.2	4.0	0.42	30	121	133	1.05	6.9 (± 1.17)
Beef/Lentils2	4.5	0.2	4.3	0.56	30	113	123	1.03	6.8 (± 1.21)
Beef2	3.6	0.4	3.2	0.64	30	98	74	1.79	6.9 (± 1.37)
Target value	4.5	N/A	N/A	0.44–0.66	23	240	N/A	N/A	N/A

Recipe	Total Iron (mg)	Heme iron	Non-heme iron	Absorbable iron (mg)	Vitamin C (mg)	Calcium (mg)	Phytate phosphorous (mg)	CO_2_e (kg/meal)
b. Complete Meal (with bulgur, salad buffet and school-standard milk)								
Soy1	5.6	0.0	5.6	0.20	46	364	508	0.47
Beef/Soy 1	5.4	0.2	5.2	0.40	46	339	467	1.33
Beef/Lentils1	6.4	0.2	6.2	0.53	46	315	456	1.31
Beef/Beans1	5.4	0.3	5.1	0.55	46	385	425	1.75
**Original	5.0	0.4	4.6	0.63	47	272	391	2.06
Beef/Soy2	6.1	0.2	5.9	0.45	47	341	484	1.34
Beef/Lentils2	6.4	0.2	6.2	0.55	47	330	474	1.32
Beef2	5.5	0.4	5.1	0.64	46	314	425	2.09
Target value	4.5	N/A	N/A	0.44–0.66	23	240	N/A	N/A

Recipe	Total Iron (mg)	Heme iron	Non-heme iron	Absorbable iron (mg)	Vitamin C (mg)	Calcium (mg)	Phytate phosphorous (mg)	CO_2_e (kg/meal)
c. Complete Meal (with bulgur, salad buffet, excluding milk)								
Soy1	5.6	0.0	5.6	0.22	46	178	508	0.35
Beef/Soy 1	5.4	0.2	5.2	0.51	46	153	467	1.21
Beef/Lentils1	6.4	0.2	6.2	0.72	46	129	456	1.19
Beef/Beans1	5.4	0.3	5.1	0.63	46	199	425	1.63
**Original	5.0	0.4	4.6	0.85	48	126	216	1.94
Beef/Soy2	6.1	0.2	5.9	0.58	46	181	484	1.22
Beef/Lentils2	6.4	0.2	6.2	0.73	46	144	474	1.20
Beef2	5.5	0.4	5.1	0.92	46	128	425	1.97
Target value	4.5	N/A	N/A	0.44–0.66	23	240	N/A	N/A

All values for complete meals include chili con carne with bulgur (150 g cooked weight), raw vegetables (1taste tests.38 g), crispbread (1 slice, 14 g), spread (10 g, 40% fat), and milk (100 mL, 1.5% fat). Absorbable iron calculated for individuals with serum ferritin <15 μg/L using the Hallberg and Hulthén (7) algorithm with the following parameters: soy protein 26 g/100 g (based on a product commonly used with in public school meals), phytate values from Hallberg and Hulthén (7) for beans and lentils, Mayer-Labba et al. (8) for soy mince, Ertaş (28) for bulgur, and calcium from Swedish Food Composition Database v.2022-05-24 (27). CO₂e values from RISE Food Climate Database (accessed 2024, mean values per product). Hedonic scores presented as mean ± SD on 9-point scale (1 = dislike extremely, 9 = like extremely). Round 1: *n* = 48 participants. Round 2: *n* = 54 participants. Target value for absorbable iron: 0.44 mg for median teenage girl, 0.66 mg for 95th percentile (12), values for calcium and vitamin C from the Swedish National Food agency (22). *For vitamin C in the algorithmic calculations cooking loses of 50% is applied as the recipe is based on raw ingredients **The original recipe is a beef mince recipe provided by a school meal kitchen, the recipe is only calculated not part of the taste tests.

When analyzing absorbable iron content, the Beef/Lentils1 recipe provided the highest levels. In contrast, the Soy1 recipe failed to meet the target in any meal combination. Beef/Lentils1 was the only optimized recipe that exceeded the recommended threshold of 0.66 mg when served as a complete meal without milk. All recipes met the reference values for energy and nutrient content for an average school lunch, corresponding to 30% of the recommended daily intake (RDI) for children aged 13–15 years, except for vitamin D in the complete meal served with milk. All recipes assessed in the first round reduced carbon footprint by 16–84% compared to the original recipe. The Soy1 recipe demonstrated the lowest carbon footprint of all meal combinations, with a reduction of 73% for the complete meal (including bulgur and standard). Conversely, the Beef/Beans1 recipe showed the least improvement, reducing CO₂e for the complete meal by only 16%. Recipes Beef/Soy1 and Beef/Lentils 1 reduced their CO₂e by 38 and 39%, respectively.

Recipe Beef/Beans1 had the highest mean hedonic value, 7.1 ± 1.3. The mean in hedonic liking for the recipes assessed in test round 1 ranged from 6.5 ± 1.6 to 7.1 ± 1.3. The Friedman’s ANOVA showed no significant difference in liking between recipes [χ^2^ (3) = 4.79, *p* = 0.188, Kendall’s W = 0.038]. Post-hoc pairwise comparisons using Wilcoxon signed-rank tests with Bonferroni correction (adjusted *α* = 0.008 for six comparisons) confirmed no significant differences between any recipe pairs (all *p* > 0.008). Although two pairwise comparisons for the richness attribute showed nominal significance at the uncorrected level (Soy1 vs. Beef/Beans1, *p* = 0.029; Beef/Lentils1 vs. Beef/Beans1, *p* = 0.014), neither survived Bonferroni correction, indicating these were likely Type I errors attributable to multiple testing.

When asked to rank their favorite variant of chili con carne, 30.4% (n = 14) of participants chose Beef/Soy1 as their preferred choice. Beef/Beans1 followed closely with 26.1% (n = 12) of the votes. Meanwhile, Soy1 and Beef/Lentils1 were equally popular, each receiving 21.7% (n = 10) of the preferences.

**Figure 1:**
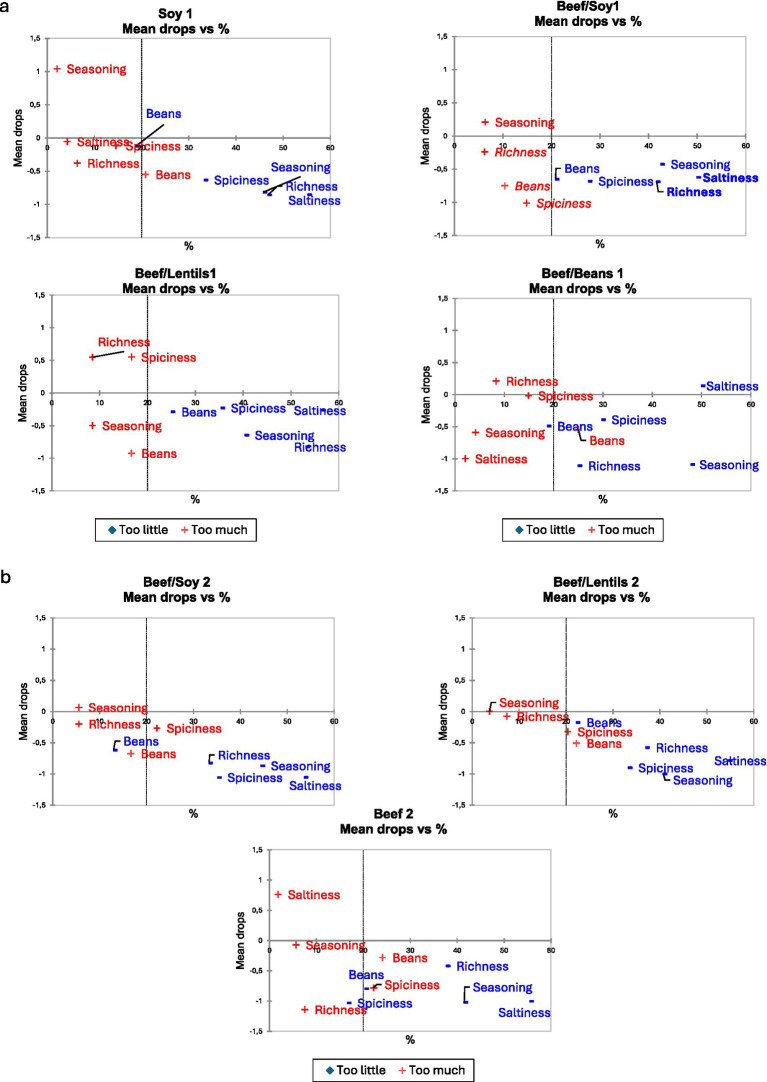
Mean drop plots for chili con carne recipe variations in test round 1 **(a)** and test round 2 **(b)**. Penalty analysis based on the ideal recipe profile. Each subplot shows a different recipe variation (labeled). The x-axis shows the percentage of consumers rating each attribute as” too little” or” too much”; the y-axis shows the mean drop in overall liking. Red markers (+) indicate ‘too little’; blue markers (◆) indicate ‘too much’. Bold text = statistically significant mean drop; italic text = statistically significant penalty. Vertical dashed line = 20% consumer threshold.

**Figure 2:**
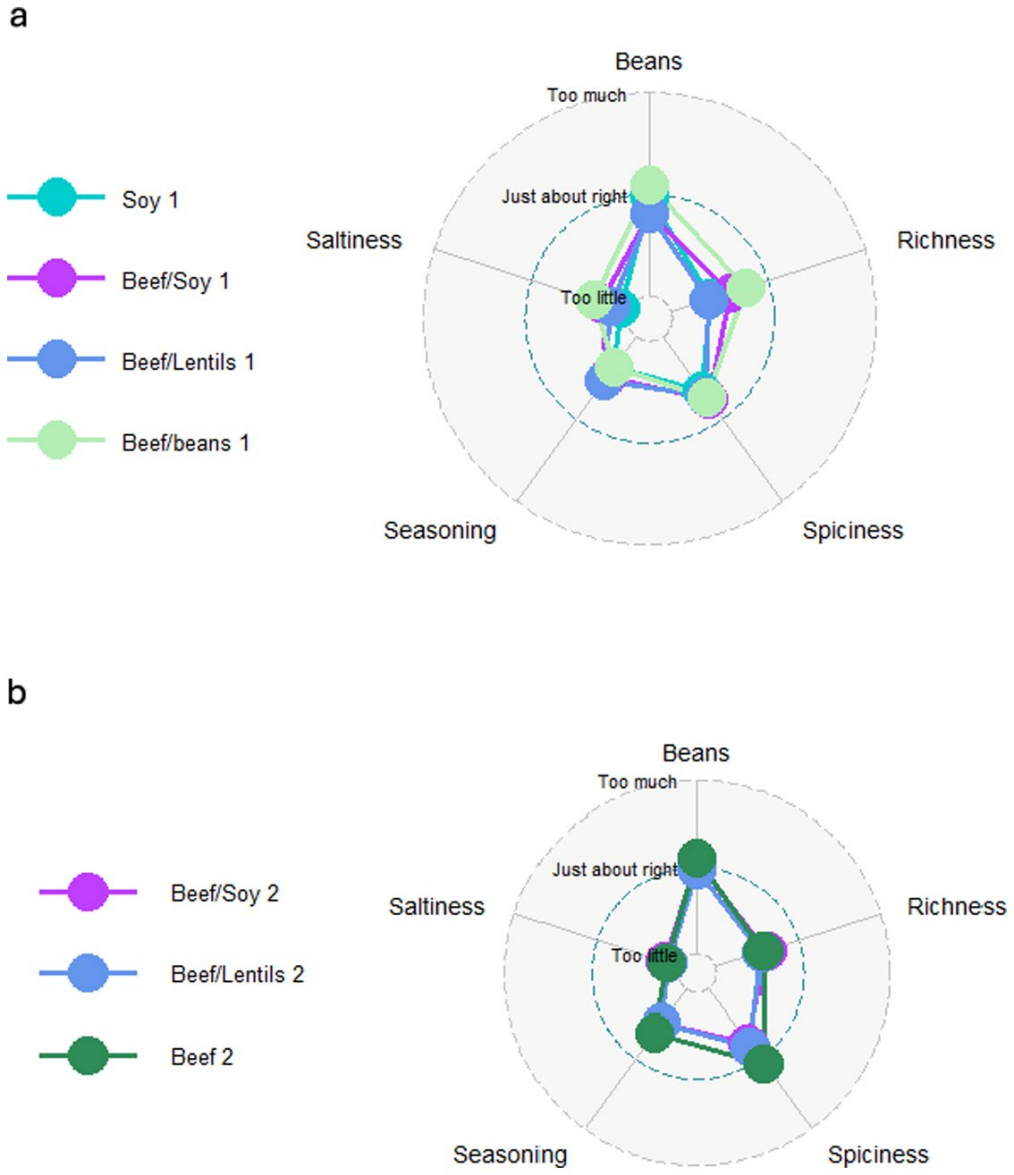
Radar charts showing Just-About-Right (JAR) scores for five sensory attributes across chili con carne recipe variations. **(a)** Test round 1; **(b)** Test round 2. Each axis represents one attribute (Beans, Richness, Spiciness, Seasoning, and Saltiness) with scores ranging from ‘Too little’ (center) through ‘Just about right’ (blue dotted line) to ‘Too much’ (outer edge). Colored lines represent different recipe formulations.

To examine how variations in sensory attributes might affect the overall liking of the samples, a modified penalty analysis was performed individually for all samples in test round 1, expressed as mean drop in overall liking, Figure 1a. Most attributes did not differ significantly in the penalty analysis, the only significant drop in liking was found in Beef/Soy1 concerning Richness (mean drop = 1.2 points, *p* = 0.03) and Saltiness (mean drop = 0.8 points, *p* = 0.04). The perception of ‘too little’ saltiness influenced the scores, but this was only significant for the Beef/Soy1 sample. The JAR scores for the five attributes across the four samples in round 1 are visualized as a radar plot in Figure 2a. The radar plot also shows small variations between the four samples.

#### 3.1.2 Round 2 - recipe refinement and validation

The second round proceeded with Beef/Soy1 and Beef/Lentils1 recipes as they provided the best balance between absorbable iron, carbon footprint and taste preference. Further, the original recipe with 50 g of ground beef that was not assessed in round 1 and was added as a sample in round 2.

Analysis of the attributes in round 1 revealed that the average decline in preference was attributed to perceptions of insufficient richness, seasoning, and saltiness, serving as key areas for taste improvement. A further improvement was the replacement of 15% fat ground beef with 10% fat ground beef to improve the lipid profile.

Based on the results of round 1, an improved recipe base was developed for all subsequent cooking elaborations. This base remained consistent, with only the meat-substitutes varying. In the cooking elaboration of round 2, we assessed variants of Beef/Soy1 and Beef/Lentils1 that included shredded mushrooms in brine, aiming to enhance texture, saltiness and umami flavor. However, these iterations were discarded due to a perceived decrease in richness. In the next step of round 2, we modified Beef/Soy1 and Beef/Lentils1 recipes by doubling the cumin content and adding a tablespoon of sun-dried tomato paste. This adjustment was found to enhance the richness, making these recipes suitable for subsequent consumer evaluations and are from here on referred to as Beef/Soy2 and Beef/Lentils2. The salt content was not increased, this disregard of the penalty analysis result was to adhere to the recommended limit of 1.8 g per school lunch for children aged 13–15 years (22).

Test samples were prepared according to the same procedure as in round 1. In round 2, we assessed two improved recipes to one like the original, using the same base seasoning to compare the effects of soy mince and lentils with ground beef. The recipes assessed in the consumer evaluations in round 2 were: Beef/Soy2 (25 g ground beef /25 g soy mince), Beef/Lentils2 (25 g ground beef/15 g lentils) and Beef2 (50 g ground beef). These recipes were assessed for their absorbable iron, energy, and nutrient content (Table 3) as well as their carbon footprint, and hedonic value (Table 2). Recipes Beef/Lentils2 and Beef2 met the basic requirement for absorbable iron for the median teenage girl of 0.44 mg per meal but only covered the 95^th^ percentile when served as a complete meal without milk. Recipe Beef/Soy2 was able to cover the needs of the median girl when served as a complete meal regardless of drink but were in no combination able to cover the 95^th^ percentile. Concerning other nutrients, just like in round 1, all recipes met the nutrient and energy content of an average school lunch except for vitamin D (Table 3).

**Table 3:** Energy, nutrients, iron and CO_2_e - test round 2.

Nutrient	Beef/Soy2 Mean ± SD	Beef/Lentils2 Mean ± SD	Beef2 Mean ± SD	% of RDI (13–15 years)
a. Chili Only (without sides)				
Energy (kcal)	297 ± 5	307 ± 5	288 ± 5	39–42%
Protein (g)	16.3 ± 1	17.0 ± 1	17.4 ± 1	65–70%
Fat (E%)	43.4	36.4	45.1	25–40% ref
- SFA (E%)	6.8	5.9	9.9	≤10% ref
- PUFA (E%)	10.9	9.3	9.1	5–10% ref
- MUFA (E%)	23.0	18.7	23.1	10–20% ref
Carbohydrates (E%)	34.3	41.2	30.5	45–60% ref
Fiber (g)	10.3 ± 1	12.8 ± 1	9.7 ± 0.8	108–142%
Vitamin C (mg)	61 ± 5	61 ± 5	58 ± 5	252–265%
Vitamin D (μg)	0.0	0.0	0.0	0%
Folate (μg)	112 ± 8	123 ± 8	113 ± 8	124–137%
Iron, total (mg)	3.7 ± 0.3	4.2 ± 0.3	3.2 ± 0.3	71–93%
b. Complete meal (with bulgur, salad buffet and school-standard milk)				
Energy (kcal)	684 ± 10	695 ± 10	675 ± 10	92–95%
Protein (g)	31.4 ± 2	32.1 ± 2	32.5 ± 2	126–130%
Fat (E%)	27.8	24.9	28.3	25–40% ref
- SFA (E%)	5.3	5.0	6.7	≤10% ref
- PUFA (E%)	6.8	6.2	6.0	5–10% ref
- MUFA (E%)	13.6	11.9	13.6	10–20% ref
Carbohydrates (E%)	43.6	56.3	52.2	45–60% ref
Fiber (g)	19.8 ± 2	22.3 ± 2	19.2 ± 2	213–248%
Vitamin C (mg)	86 ± 7	86 ± 7	84 ± 7	365–374%
Vitamin D (μg)	2.5 ± 0.2	2.5 ± 0.2	2.5 ± 0.2	83%
Folate (μg)	206 ± 15	218 ± 15	208 ± 15	229–242%
Iron, total (mg)	5.7 ± 0.4	6.2 ± 0.4	5.2 ± 0.4	116–138%
c. Complete Meal (with bulgur, salad buffet, excluding milk)				
Energy (kcal)	625 ± 10	636 ± 10	617 ± 10	84–87%
Protein (g)	26.0 ± 2	26.7 ± 2	27.1 ± 2	104–108%
Fat (E%)	29.3	26.2	29.9	25–40% ref
- SFA (E%)	5.2	4.8	6.6	≤10% ref
- PUFA (E%)	7.4	6.7	6.6	5–10% ref
- MUFA (E%)	14.7	12.8	14.6	10–20% ref
Carbohydrates (E%)	53.8	56.8	52.3	45–60% ref
Fiber (g)	19.8 ± 2	22.3 ± 2	19.2 ± 2	213–248%
Vitamin C (mg)	85 ± 7	85 ± 7	83 ± 7	361–370%
Vitamin D (μg)	1.0 ± 0.1	1.0 ± 0.1	1.0 ± 0.1	33%
Folate (μg)	184 ± 12	196 ± 12	186 ± 12	204–218%
Iron, total (mg)	5.7 ± 0.4	6.2 ± 0.4	5.2 ± 0.4	116–138%

Reference values for the energy and nutrient content in an average school lunch, corresponding to 30% of the recommended daily intake (RDI) for girls and boys aged 13–15, according to the Nordic Nutrition Recommendations 2012, as stated by the National guidelines for school meals. Notes: SD values are estimated based on typical variation in recipe preparation and ingredient variation. RDI percentages are calculated based on 30% of daily recommended intake for 13–15 year olds. *E% including glycaemic carbohydrates and fiber **Only glycaemic carbohydrates.

Both recipes including meat-substitutes had a lower CO_2_e than the original recipe, Beef/Soy2 37% and Beef/Lentils2 38%. Beef2 in contrast had a slightly higher CO_2_e (1.5%) due to the changes in the base recipe, i.e., adding sun-dried tomato paste and ground beef 10% fat instead of 15%.

The mean hedonic values for round 2 were: Beef/Soy2 7.0 ± 1.4, Beef/Lentils2 6.8 ± 1.5, and Beef2 7.0 ± 1.3. The Friedman’s ANOVA showed no difference in hedonic value between the samples [χ^2^ (2) = 1.46, *p* = 0.482, Kendall’s W = 0.015]. The very low Kendall’s W value indicates minimal agreement among participants regarding preferences.

When ranking their favorite variant 40.4% (*n* = 21) they chose Beef/Soy2, 34.6% (*n* = 18) Beef2 and 25% (*n* = 13) Beef/Lentils2.

The penalty analysis showed that no sample had a statistically significant drop in mean liking based on amount of beans, richness, spiciness, seasoning or saltiness (all *p* > 0.05) (Figure 1b). Figure 2b visualizes the JAR scores for the five attributes across the three samples in round 2 as a radar plot, showing an even tighter clustering of JAR scores compared to Round 1. Notably, JAR scores for richness improved from Round 1 to Round 2: Beef/Soy increased from 52 to 61% rating it as just-about-right, and Beef/Lentils increased from 38 to 56%, indicating that the recipe modifications (increased cumin and sun-dried tomato paste) successfully enhanced the perceived richness without compromising other attributes. This suggests that taste was not a decisive factor or trade-off in these recipes.

## 4 Discussion

This study addresses critical nutritional concerns, such as absorbable iron, and taste preference in climate-adapted meals. The findings underscore that, due to the limited bioavailability of iron in current plant-based meat alternatives, trade-offs are unavoidable when adapting meals to meet both nutritional and climate goals. The developed methodology offers a structured approach to creating school meal recipes by identifying key ingredients suitable for improvement.

### 4.1 Consumer evaluation and taste preferences

In round 1, the objective was to maintain the taste of the test dishes as consistent as possible while improving other aspects such as CO_2_e and absorbable iron. None of the recipes in round 1 were found to deviate significantly in taste, indicating that no taste trade-offs were necessary. This allowed the study to focus on improving the other aspects without compromising taste.

The preference for Beef/Soy1 and Beef/Lentils1 recipes in round 1, as well as Beef/Soy2 in round 2, suggests that hybrid recipes are both acceptable and appealing to consumers. Hybrid recipes have been found generally well-received, even if they are perceived as lacking in meat-like flavor (33). Low meat-like flavor is often reflecting an absence of umami (34) and in the current study, the absence of umami was reflected in low JAR scores for richness. To address this, mushrooms were included in the cooking elaborations in round 2 to add texture and umami flavor. However, this did not improve the recipe, despite studies indicating that consumers accept meat-mushroom blends, as mushrooms enhance umami flavor and increase savoriness (34). Further adjustments were made such as increasing cumin and adding sun-dried tomato paste, to enhance the sensory attributes. Sun-dried tomatoes contain glutamine, known to increase umami taste (35).

Improvements in JAR scores for richness were observed in round 2, with scores increasing from 52 to 61% for Beef/Soy2 and from 38 to 56% for Beef/Lentils2, indicating that the adjustments had the desired effect. The study utilized hybrid recipes with soy mince or lentils, but the current market offers a wide variety of plant-based minces and other legumes, creating opportunities for many different hybrid combinations. Baune et al. (33) found that hybrid recipes combining pea protein with ground pork were the most popular, highlighting pea protein’s potential to enhance the sensory appeal of such dishes. Given these findings, it would be valuable to test pea protein in recipes in the future to determine if it can improve hedonic ratings. Additionally, certain pea protein cultivars have demonstrated a decreased phytate content (36).

### 4.2 Nutritional considerations and iron bioavailability

The results underscore the challenge of iron bioavailability in plant-based meals. Our findings align with previous studies (8, 11) that highlight the limitations of plant-based iron sources. Despite the high total iron content, the bioavailability of the purely plant-based Soy1 and the hybrid beef-soy recipes consistently failed to meet the absorbable iron requirements for teenage girls with the highest needs. In some instances, they did not even fulfill the average requirement or the school lunch target of 30% of daily iron needs (12). These findings are troubling as public-school meals in Sweden often rely on replacing meat with soy alternatives to reduce CO_2_e emissions (21).

To address nutritional trade-offs, one can offset nutrient loss from red meat reduction by increasing climate-friendly foods like lentils (37). Still, this approach does not consider the issue of absorbable iron. In fact, no study on the optimization of school meals has previously recognized the issues of iron bioavailability when increasing the proportion of plant-based alternatives. To our knowledge, optimization efforts so far have focused solely on evaluating total iron content, even though Colombo et al. (37) set higher iron requirements for vegetarian meals. The current study highlights this by demonstrating the discrepancy between total iron and absorbable iron. While total iron content might appear sufficient, the current study shows that levels of absorbable iron often fall short of desired standards (12). In Soy1 the total iron content was well within recommended levels when served as a complete meal, but absorbable iron did not reach the desired levels in any meal combination. Upon closer examination, absorbable iron only reached 35% of the requirement for the 95th percentile (12) emphasizing that the trade-off becomes unacceptable. Further, a study, found that girls having dietary habits with the lowest climate impact had more than twice the risk of iron deficiency compared to those with the highest climate impact (13). This nutritional challenge is also evident in dietary patterns, that vegetarians/vegans exhibited the highest rates of iron deficiency, followed by pescatarians, while omnivores maintained substantially higher ferritin levels (38).

Iron deficiency and anemia in teenagers can have significant implications on physical health, cognitive development, and overall well-being (39). Since iron is crucial for growth and development, deficiency during adolescence, a period characterized by rapid growth, can lead to delayed physical development (16, 40). Cognitively, iron deficiency during adolescence can impair essential functions such as attention, memory, and learning abilities (15). Consequently, this can affect academic performance, leading to struggles in school and impact educational attainment (15, 41).

### 4.3 Balancing sustainability and nutrition

Compared to the nutritional guidelines for school meals, all recipes met the recommended nutritional values for adolescents aged 13–15 years (22) when served as a complete meal, except for vitamin D, which reached 83% of the recommended level. Notably, our focus was on developing a method to improve selected recipes rather than an entire menu and nutrients like vitamin D have a large day to day variation. One of the significant outcomes of this study is the development of recipes that reduce carbon footprint while maintaining or enhancing nutritional value. Consistent with previous research on Swedish school meals (37, 42) our recipe optimization reduced CO_2_e emissions substantially (16–84%).

All optimized recipes successfully reduced their carbon footprint, recipe Soy1 showed the greatest improvement (84% reduction). Further, neither recipe Beef/Lentils1, Beef/Beans1 nor Beef/Lentils2 yielded the lowest CO_2_e but demonstrated an improvement over the original recipe, while achieving an acceptable level of absorbable iron. Conversely, hybrid recipes that combined soy and meat achieved better kg CO_2_e but fell short in absorbable iron. This finding further support the argument for a moderate use of animal products to enhance nutrient absorption in predominantly plant-based diets (10), preferably incorporating plant-based alternatives other than soy.

A key method to enhance iron absorption is through strategic food pairings, such as combining plant-based foods with small amounts of meat and vitamin C. For example, incorporating vitamin C-rich foods like bell peppers alongside iron-rich options such as lentils and beans can significantly improve iron uptake. Additionally, reducing phytate-rich foods by partially substituting whole grains with refined grains and increasing energy intake from animal-sourced foods (10), may be beneficial, although this approach contradicts the NNR 2023 guidelines (Nordic Council of Ministers 2023).

In addition to food pairings, certain food processing techniques can also improve nutrient bioavailability. Soaking grains, beans, and seeds before cooking can help reduce phytates (9). Similarly, sprouting these foods can further break down phytates and increase bioavailable iron and other essential nutrients (9). Fermentation is another beneficial technique; fermented foods like tempeh and miso undergo processes that reduce phytates and enhance the bioavailability of minerals, including iron (43).

Though it is crucial to adapt our food consumption patterns to mitigate climate change (5), this study highlights significant health trade-offs, particularly for adolescent girls aged 13–15 years. Current plant-based diets, though beneficial for reducing CO_2_e, fall short in providing an adequate amount of absorbable iron. This shortfall is particularly concerning for teenage girls, who are already at a higher risk of iron deficiency and iron deficiency anemia, globally and in Sweden (13, 44). The Agenda 2030 Sustainable Development Goals 3, 4, and 5 advocate for ensuring healthy lives, quality education, and gender equality (1), which may be difficult to achieve if iron bioavailability is not taken into account.

While promising, newly developed meat-substitutes need nutritional improvements, particularly in terms of iron bioavailability and salt content (8). There is a potential risk that if school meal planners keep addressing iron needs using total iron content of school lunch menus, the iron requirements of those with highest needs will not be met. In an eagerness to reduce the carbon footprint of food, there is a risk of sacrificing the health of a vulnerable population. This approach is neither fair nor sustainable.

### 4.4 Methodology for sustainable school meal recipes

**Figure 3:**
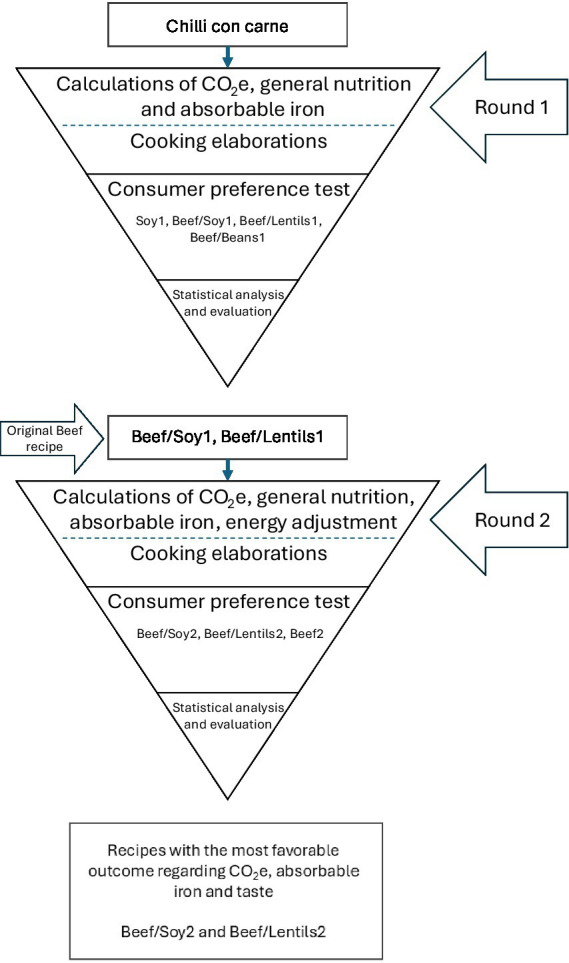
Two-stage modified culinary funnel for developing sustainable chili con carne recipes. Each stage includes environmental/nutritional calculations (CO₂e = carbon footprint), cooking trials, consumer testing, and evaluation. Round 1 evaluated four formulations; Round 2 refined the top two performers, resulting in Beef/Soy2 and Beef/Lentils2 as the optimal recipes balancing sustainability, nutrition, and taste.

To address these nutritional challenges, it is pivotal to emphasize the methodology for creating sustainable school meal recipes, as consumer acceptance is key to successfully implementing new climate-adapted options. While there may be initial reluctance toward unfamiliar foods, building familiarity through iterative testing and feedback can significantly enhance consumer acceptance (18, 45, 46). Adolescence can be accessible for changes in eating behaviors (45), making school lunches a valuable opportunity to introduce sustainable foods. Our iterative approach ensures continuous refinement and enhancement of school meal recipes, balancing nutritional needs, carbon footprint, and taste preferences. As illustrated in Figure 3, our methodological approach employed a culinary funnel process (18) that systematically narrowed down recipe options while optimizing for multiple factors simultaneously.

This adaptation of the culinary funnel principle allowed us to start with a traditional recipe and progressively refine it through controlled variations, rather than beginning with specific crop varieties as in Westling et al.’s (18) original framework. The visual representation highlights how the process integrates quantitative assessments (nutritional analysis, absorbable iron content, CO₂e calculations) with qualitative evaluations (cooking elaborations, consumer preference tests) across multiple iterations, following the iterative testing approach recommended by Westling et al. (18). This systematic narrowing approach not only produced recipes that balanced nutritional, environmental, and sensory considerations but also documented a replicable methodology that school meal planners could adopt for improving other recipes.

Consumer evaluations and taste preference tests play a crucial role in this process, providing valuable feedback to improve recipe acceptance (46). This feedback loop ensures that the final recipes are not only nutritionally adequate but also appealing to consumers. However, an important limitation of this study must be acknowledged: there is a fundamental mismatch between our evaluation population and target audience.

## 5 Strengths and limitations

While our methodology was designed specifically for adolescent girls and boys aged 13–15 years, a nutritionally vulnerable group with high iron requirements, the sensory evaluations were conducted with university staff and students ranging from ages 19 to 66 years. This decision was made due to the complexity and length of the sensory protocols employed (15–25-min sessions with multiple rating scales), which would have been challenging to implement in a school setting during limited lunch periods. Taste preferences and food acceptance patterns differ significantly between adults and adolescents. Adolescents typically show greater sensitivity to bitter tastes, stronger preferences for familiar flavors, and different thresholds for texture acceptance compared to adults (19). Additionally, the social context of eating, consuming meals in a school cafeteria with peers versus in a controlled university testing environment, can substantially influence food acceptance and consumption patterns. Peer influence, time constraints, and the broader meal context in schools are factors that were not captured in our adult evaluation setting. Therefore, while our results successfully demonstrate that hybrid recipes can be optimized for multiple factors simultaneously (iron bioavailability, carbon footprint, and sensory attributes) in a controlled laboratory setting, direct extrapolation of the acceptability findings to adolescent populations is not scientifically justified. The hedonic ratings and preference rankings we obtained reflect adult perceptions and cannot be assumed to represent how teenage girls would rate these same recipes. Our methodology validates the technical approach to balancing nutritional, environmental, and sensory factors, but the specific recipes we developed require further validation.

The essential next step is validation of these recipes in school canteens with the target adolescent population. This validation should employ simplified sensory protocols appropriate for school settings, such as brief preference rankings or simple yes/no acceptability questions that can be completed within the constraints of a school lunch period. Such protocols, while less detailed than the 9-point hedonic scales and JAR analyses we employed, are more ecologically valid and can provide actionable information about whether these recipes would be accepted in practice (46).

More accessible evaluators, such as university affiliates, are commonly used early in product development to identify innovative recipes and preparation methods that may appeal to a broader market before conducting expensive large-scale testing (46). Our study represents this preliminary development phase. The developed methodology, systematically assessing absorbable iron, CO₂e, and sensory qualities, provides a framework adaptable to school settings. School meal planners can use this approach to develop and refine recipes, though final acceptability testing must occur with the target population. While our specific recipes require validation with adolescents in schools, we have demonstrated a replicable methodology for balancing sustainability, nutrition, and acceptability in meals for vulnerable populations.

Beyond the evaluator mismatch discussed above, several additional methodological considerations merit attention. The choice of algorithm was based on previous literature (De (6, 7, 23, 24)) where it was found to be the most accurate for our intended population. This algorithm is the only one available that considers interaction between enhancers and inhibitors but has been criticized because it is based on “single meal studies.” However, when designing the algorithm it was validated with a 10-day experiment (7, 47) Further, absorbable iron calculated here is unlikely to be over-estimated since iron absorption is downregulated when iron stores are adequate, and upregulated when iron stores are low (7). The algorithm allows calculations in relation to iron stores, and we chose to perform the calculations for an individual with low or empty iron stores (serum ferritin 15 μg/L) (7). This means that the absorbable iron is already calculated to be upregulated compared to an iron replete individual.

Another factor to consider is phytate content in the different foods and how it was analyzed. Since comprehensive information on phytate fractions is missing, values were gathered from the literature (7–9, 28) for all the ingredients in the recipes. Often, this information represents an average value for a type of product rather than the specific value of the exact product used in the recipe. Further, the data is built on analyses of different fractions of phytate. However, iron bioavailability is crucial to include when climate adapted meals are planned for population groups with the highest iron needs, and we consider the tools and values used here to be the best available. Phytate values were derived from literature rather than direct measurement, which may introduce some variability due to differences in cultivars or growing conditions.

The culinary funnel methodology imposes analytical constraints by preventing direct statistical comparisons across test rounds, requiring separate evaluation of each iteration despite recipe similarities. This approach increases complexity and may overlook optimal combinations outside the refinement pathway. However, the methodology provides substantial benefits through systematic iteration, enabling targeted recipe improvements. The indistinguishable flavor profiles among Round 2 recipes demonstrates methodological success, establishing a robust flavor foundation that allowed decision-making to focus on CO_2_e and iron bioavailability while maintaining consistent sensory quality.

Additionally, the calculations use a specific portion size and standard that reflect what is offered, not what is consumed. This standard includes around 30% of the recommended daily intake of vitamin C, which positively affects iron absorption. However, if the portion size is not consumed as intended, this benefit might be reduced. Nonetheless, the legal requirement is to provide a nutritionally adequate meal.

## 6 Conclusion

This study of variants of chili con carne demonstrates that systematic recipe optimization using an adapted culinary funnel methodology can effectively balance nutritional adequacy, environmental sustainability, and consumer acceptance in school meal planning. Through iterative testing and refinement, hybrid recipes achieved carbon footprint reductions of 37–39% while maintaining adequate iron bioavailability and consumer appeal, demonstrating that compromises between health and climate-adaptation are not inevitable when recipe development is strategically approached.

Critically, this research reveals that evaluating absorbable iron, rather than total iron content, is essential when developing plant-based meals for vulnerable populations, particularly adolescent girls. This study provides school meal planners and researchers with a practical framework for creating recipes that protect both planetary and human health. While our specific recipes were evaluated with adults in a laboratory setting and require validation with adolescent populations in school environments, the systematic approach we developed could inform future school meal planning. Pending such validation, this methodology provides a framework for ensuring that the shift toward climate-adapted school meals does not compromise the taste preferences or health and well-being of students. The essential next step is to test these recipes with the target adolescent population in school canteens using simplified, age-appropriate sensory protocols to confirm their acceptability and consumption in real-world settings.

## Data Availability

The datasets presented in this study can be found in online repositories. The names of the repository/repositories and accession number(s) can be found at: https://researchdata.se/en/catalogue/dataset/2024-413.
