# Supplementary material for: Balancing trade-offs between nutritional quality, consumer acceptability and climate impact across a spectrum of chili con carne formulations: from plant-based to hybrid
Source: Front Nutr. 2025 Nov 21;12:1716322. doi: 10.3389/fnut.2025.1716322 (PMC12678115; doi:10.3389/fnut.2025.1716322)
Supplement: Supplementary file 1 [file Data_Sheet_1.pdf]

Supplemental Material 1

Recipes Test Round 1 and 2

Recipes Test Round 1

Soy 1

Soy 1	1 serving	4 servings
Yellow onion		100 g
Bell pepper		150 g
Soy mince		200 g
Carrot		100 g
Celeriac		80 g
Crushed tomatoes		400 g
Lima beans		115 g
Kidney beans		115 g
Rapeseed oil		32 g
Garlic cloves		2 pcs (10g)
Tomato paste		1 tbsp (18g)
Paprika powder		1 tbsp (6g)
Cumin		1 tsp (2g)
Cayenne pepper		0.5 tsp (2g)
Bouillon cube		0.5 pc (11g)
Salt and pepper		
PRICE	15.23 SEK	60.93 SEK

Instructions:

1. Blend yellow onion, carrot and celeriac and fry over medium heat in rapeseed oil. Finely chop garlic.

2. Add the mince and fry together.

3. Add garlic, spices and tomato paste and fry for a while longer.

4. Pour in crushed tomatoes, bouillon and a little water and let simmer for about 10 minutes.

5. Drain the beans, chop the bell pepper and add to the pot together with the beans and let simmer for about 10 more minutes.

Beef/Soy 1

Beef/Soy 1	1 serving	4 servings
Yellow onion		100 g
Bell pepper		150 g
Soy mince		100 g
Ground beef		100 g
Carrot		100 g
Celeriac		80 g
Crushed tomatoes		400 g
Lima beans		115 g
Kidney beans		115 g
Rapeseed oil		32 g
Garlic cloves		2 pcs
Tomato paste		1 tbsp
Paprika powder		1 tbsp
Cumin		1 tsp
Cayenne pepper		0.5 tsp
Bouillon cube		0.5 pc
Salt and pepper		
PRICE	16.13 SEK	64.54 SEK

Instructions:

1. Blend yellow onion, carrot and celeriac and fry over medium heat in rapeseed oil. Finely chop garlic.

2. Add the mince and fry together.

3. Add garlic, spices and tomato paste and fry for a while longer.

4. Pour in crushed tomatoes, bouillon and a little water and let simmer for about 10 minutes.

5. Drain the beans, chop the bell pepper and add to the pot together with the beans and let simmer for about 10 more minutes.

Beef/Lentils 1

Beef/Lentils 1	1 serving	4 servings
Yellow onion		100 g
Bell pepper		150 g
Ground beef		100 g
Red lentils (dried)		60 g
Carrot		100 g
Celeriac		80 g
Crushed tomatoes		400 g
Lima beans		115 g
Kidney beans		115 g
Rapeseed oil		32 g
Garlic cloves		2 pcs
Tomato paste		1 tbsp
Paprika powder		1 tbsp
Cumin		1 tsp
Cayenne pepper		0.5 tsp
Bouillon cube		0.5 pc
Salt and pepper		
PRICE	14.78 SEK	59.13 SEK

Instructions:

1. Blend yellow onion, carrot and celeriac and fry over medium heat in rapeseed oil. Finely chop garlic.

2. Add the mince and fry together.

3. Add garlic, spices and tomato paste and fry for a while longer.

4. Add lentils, crushed tomatoes, bouillon and a little water and let simmer for about 10 minutes.

5. Drain the beans, chop the bell pepper and add to the pot together with the beans and let simmer for about 10 more minutes.

Beef/Beans 1

Beef/Beans 1	1 serving	4 servings
Yellow onion		100 g
Bell pepper		150 g
Ground beef		120 g
Carrot		100 g
Celeriac		80 g
Crushed tomatoes		400 g
Lima beans		170 g
Kidney beans		170 g
Rapeseed oil		32 g
Garlic cloves		2 pcs
Tomato paste		1 tbsp
Paprika powder		1 tbsp
Cumin		1 tsp
Cayenne pepper		0.5 tsp
Bouillon cube		0.5 pc
Salt and pepper		
PRICE	16.12 SEK	64.48 SEK

Instructions:

1. Blend yellow onion, carrot and celeriac and fry over medium heat in rapeseed oil. Finely chop garlic.

2. Add the mince and fry together.

3. Add garlic, spices and tomato paste and fry for a while longer.

4. Pour in crushed tomatoes, bouillon and a little water and let simmer for about 10 minutes.

5. Drain the beans, chop the bell pepper and add to the pot together with the beans and let simmer for about 10 more minutes.

Recipes Test Round 2

Beef/Soy 2

Beef/Soy 2	1 serving	4 servings
Yellow onion		100 g
Bell pepper		150 g
Soy mince		100 g
Ground beef		100 g
Carrot		100 g
Celeriac		80 g
Crushed tomatoes		400 g
Lima beans		115 g
Kidney beans		115 g
Rapeseed oil		32 g
Garlic cloves		2 pcs
Tomato paste		1 tbsp
Sun-dried tomato cream		1 tbsp
Paprika powder		1 tbsp
Cumin		1 tsp
Cayenne pepper		0.5 tsp
Bouillon cube		1 pc
Salt and pepper		
PRICE	16.13 SEK	64.54 SEK

Instructions:

1. Blend yellow onion, carrot and celeriac and fry over medium heat in rapeseed oil. Finely chop garlic.

2. Add the mince and fry together.

3. Add garlic, spices and tomato paste and fry for a while longer.

4. Pour in crushed tomatoes, bouillon and a little water and let simmer for about 10 minutes.

5. Drain the beans, chop the bell pepper and add to the pot together with the beans and let simmer for about 10 more minutes.

Beef/Lentils 2

Beef/Lentils 2	1 serving	4 servings
Yellow onion		100 g
Bell pepper		150 g
Ground beef		100 g
Red lentils (dried)		60 g
Carrot		100 g
Celeriac		80 g
Crushed tomatoes		400 g
Lima beans		115 g
Kidney beans		115 g
Rapeseed oil		32 g
Garlic cloves		2 pcs
Tomato paste		1 tbsp
Sun-dried tomato cream		1 tbsp
Paprika powder		1 tbsp
Cumin		1 tsp
Cayenne pepper		0.5 tsp
Bouillon cube		1 pc
Salt and pepper		
PRICE	14.78 SEK	59.13 SEK

Instructions:

1. Blend yellow onion, carrot and celeriac and fry over medium heat in rapeseed oil. Finely chop garlic.

2. Add the mince and fry together.

3. Add garlic, spices and tomato paste and fry for a while longer.

4. Add lentils, crushed tomatoes, bouillon and a little water and let simmer for about 10 minutes.

5. Drain the beans, chop the bell pepper and add to the pot together with the beans and let simmer for about 10 more minutes.

Beef 2

Beef 2	1 serving	4 servings
Yellow onion		100 g
Bell pepper		150 g
Ground beef		200 g
Carrot		100 g
Celeriac		80 g
Crushed tomatoes		400 g
Lima beans		115 g
Kidney beans		115 g
Rapeseed oil		32 g
Garlic cloves		2 pcs
Tomato paste		1 tbsp
Paprika powder		1 tbsp
Cumin		1 tsp
Cayenne pepper		0.5 tsp
Bouillon cube		1 pc
Salt and pepper		
PRICE	16.13 SEK	64.54 SEK

Instructions:

1. Blend yellow onion, carrot and celeriac and fry over medium heat in rapeseed oil. Finely chop garlic.

2. Add the mince and fry together.

3. Add garlic, spices and tomato paste and fry for a while longer.

4. Pour in crushed tomatoes, bouillon and a little water and let simmer for about 10 minutes.

5. Drain the beans, chop the bell pepper and add to the pot together with the beans and let simmer for about 10 more minutes.
