# Supplementary material for: Balancing trade-offs between nutritional quality, consumer acceptability and climate impact across a spectrum of chili con carne formulations: from plant-based to hybrid
Source: Front Nutr. 2025 Nov 21;12:1716322. doi: 10.3389/fnut.2025.1716322 (PMC12678115; doi:10.3389/fnut.2025.1716322)
Supplement: Supplementary file 2 [file Data_Sheet_2.pdf]

Supplementary Material 2: Baseline content of iron, inhibitors, and enhancers in individual ingredients

Ingredient	Iron (mg/100g)	*Phytate phosphorus (mg/100g)	Calcium (mg/100g)	Vitamin C (mg/100g)	Reference
Minced beef	2.5 (40% heme iron)	N/A	12	N/A	Swedish National Food database *Hallberg and Hulthén. 2000
Lentils	2.7	120	10	N/A	Swedish National Food database *Hallberg and Hulthén. 2000
Texturized soy protein	2.5	580	110	N/A	Swedish National Food database *Mayer-Labba et al. 2022.
Onion	0.2	16	31	8	Swedish National Food database *Hallberg and Hulthén. 2000
Red peppers	0.3	0.5	8	144	Swedish National Food database 2000
Kidney bean	1.9	271	79	N/A	Swedish National Food database *Hallberg and Hulthén. 2000
Lima beans	2.7	269	50	N/A	Swedish National Food database *Hallberg and Hulthén. 2000
Garlic	1.0	4	19	13	Swedish National Food database *Hallberg and Hulthén. 2000
Tomato paste	1.4	2	45	43	Swedish National Food database *Hallberg and Hulthén. 2000
Sun dried tomato paste	2.1	3	68	65	Swedish National Food database *Hallberg and Hulthén. 2000
Crushed tomatoes	0.6	2	12	15	Swedish National Food database *Hallberg and Hulthén. 2000

Values are presented per 100 g of edible portion. Iron values include both heme and non-heme iron, with the proportion of heme iron specified for animal-based ingredients where available. Phytate phosphorus values are used as an indicator of total phytate content. “N/A” indicates that data were not available in the referenced sources. Nutrient data were primarily obtained from the Swedish National Food Database, complemented with additional values from Hallberg and Hulthén (2000) and Mayer-Labba et al. (2022) as indicated.
