# Supplementary material for: Balancing trade-offs between nutritional quality, consumer acceptability and climate impact across a spectrum of chili con carne formulations: from plant-based to hybrid
Source: Front Nutr. 2025 Nov 21;12:1716322. doi: 10.3389/fnut.2025.1716322 (PMC12678115; doi:10.3389/fnut.2025.1716322)
Supplement: Supplementary file 3 [file Data_Sheet_3.pdf]

Supplemental Material 4: Participant Characteristics

Table S1. Demographic characteristics of participants in sensory evaluation rounds

Characteristic	Round 1	Round 2	Total Unique
Total participants	48	54	92
Age range (years)	21-65	19-66	19-66
**Gender distribution, n (%)**			
Women	32 (66.7%)	32 (59.3%)	58 (63.0%)
Men	15 (31.3%)	21 (38.9%)	33 (35.9%)
Gender undisclosed	1 (2.1%)	1 (1.9%)	1 (1.1%)
Number of evaluation sessions	5	4	9
Participants per session (range)	7-12	11-21	7-21
Participants in both rounds	-	-	10
Participant type	University staff and students		
Dietary pattern	Omnivores (self-reported)		

Notes:

All participants provided written informed consent and confirmed absence of food allergies or sensitivities. Total unique participants = 92 (48 in Round 1 + 54 in Round 2 - 10 who participated in both rounds). Evaluations were conducted at the University of Gothenburg between spring 2023 (Round 1) and spring 2024 (Round 2). The study was approved by the Swedish Ethical Review Authority (No. 2022-04834-01).
