# Supplementary material for: Balancing trade-offs between nutritional quality, consumer acceptability and climate impact across a spectrum of chili con carne formulations: from plant-based to hybrid
Source: Front Nutr. 2025 Nov 21;12:1716322. doi: 10.3389/fnut.2025.1716322 (PMC12678115; doi:10.3389/fnut.2025.1716322)
Supplement: Supplementary file 4 [file Data_Sheet_4.pdf]

Supplementary material 3

Bonferroni Correction for Round 1

Pairwise Comparisons: Soy1, BeefSoy1, BeefLentils1, BeefBeans1

Comparison	Attribute	p-value	Sig (α=0.05)	Sig (Bonf)
Soy1 vs BeefSoy1	Beans	0.295826	No	No
Soy1 vs BeefSoy1	Richness	0.326448	No	No
Soy1 vs BeefSoy1	Heat	0.583393	No	No
Soy1 vs BeefSoy1	Spiciness	0.541844	No	No
Soy1 vs BeefSoy1	Saltiness	0.286176	No	No
Soy1 vs BeefLentils1	Beans	0.394079	No	No
Soy1 vs BeefLentils1	Richness	0.818720	No	No
Soy1 vs BeefLentils1	Heat	0.828091	No	No
Soy1 vs BeefLentils1	Spiciness	0.384759	No	No
Soy1 vs BeefLentils1	Saltiness	0.637215	No	No
Soy1 vs BeefBeans1	Beans	0.668595	No	No
Soy1 vs BeefBeans1	Richness	0.029079	**Yes**	No
Soy1 vs BeefBeans1	Heat	0.651164	No	No
Soy1 vs BeefBeans1	Spiciness	0.965055	No	No
Soy1 vs BeefBeans1	Saltiness	0.176494	No	No
BeefSoy1 vs BeefLentils1	Beans	0.949026	No	No
BeefSoy1 vs BeefLentils1	Richness	0.193774	No	No
BeefSoy1 vs BeefLentils1	Heat	0.715007	No	No
BeefSoy1 vs BeefLentils1	Spiciness	0.795468	No	No
BeefSoy1 vs BeefLentils1	Saltiness	0.485154	No	No
BeefSoy1 vs BeefBeans1	Beans	0.151544	No	No
BeefSoy1 vs BeefBeans1	Richness	0.142253	No	No
BeefSoy1 vs BeefBeans1	Heat	0.909783	No	No
BeefSoy1 vs BeefBeans1	Spiciness	0.518619	No	No

Comparison	Attribute	p-value	Sig (α=0.05)	Sig (Bonf)
BeefSoy1 vs BeefBeans1	Saltiness	0.777269	No	No
BeefLentils1 vs BeefBeans1	Beans	0.217578	No	No
BeefLentils1 vs BeefBeans1	Richness	0.014337	**Yes**	No
BeefLentils1 vs BeefBeans1	Heat	0.795772	No	No
BeefLentils1 vs BeefBeans1	Spiciness	0.371128	No	No
BeefLentils1 vs BeefBeans1	Saltiness	0.322332	No	No

Total comparisons: 30 (6 pairwise comparisons × 5 attributes)

Original significance level (α): 0.05

Bonferroni corrected α: 0.001667 (0.05/30)
